# Bioremediation of hexamine and formaldehyde using a radio-tolerant bacterial consortium for efficient pollutant and COD removal

**DOI:** 10.3389/fmicb.2026.1753858

**Published:** 2026-02-10

**Authors:** S. K. Wasim Ahmed, Shaon Ray Chaudhuri

**Affiliations:** Microbial Technology Laboratory, Department of Microbiology, Tripura University, Agartala, Tripura, India

**Keywords:** ammonia, chemical oxygen demand (COD), consortium, formaldehyde, hexamine

## Abstract

**Introduction:**

Hexamine and its by-products (formaldehyde and ammonia) are classified as organic pollutants. Formaldehyde has antibacterial properties, while increased ammonia levels contribute to eutrophication, both of which disrupts microbial communities in aquatic ecosystems. Hexamine is considered a common pollutant released from various industries.

**Methods:**

Culture based analysis in enriched medium resulted in development of bacterial consortium using environmental isolates.

**Results:**

A consortium, containing six pure bacterial isolates {SRCHD03 & SRCHD04 (*Brevundimonas diminuta*), SRCHD05 & SRCHD07 (*Brucella pseudintermedia*), SRCHD06 (*Ochrobactrum sp.*), and SRCHD02 (*Micrococcus luteus*)} showed the ability to remove 1.26 g/kg hexamine associated with 0.87 g/kg of formaldehyde and 185.26 g/kg of Chemical Oxygen Demand (COD) at 96 hours of incubation under immobilized condition after 5.25 Gray of 60Co γ irradiations, starting from an initial concentration of 50 mg/L hexamine and 13367 mg/L COD, hence reporting the first radio tolerant consortium for hexamine removal.

**Conclusion:**

Involvement of *Brucella pseudintermedia* in hexamine and formaldehyde removal is being reported for the first time. The radio tolerant consortium in biofilm-based system showed enhanced hexamine, formaldehyde and COD removal efficiency compared to its suspended counterpart. This is a potential microbial formulation for the removal of hexamine and formaldehyde from stimulated wastewater.

## Graphical abstract

**Figure:**
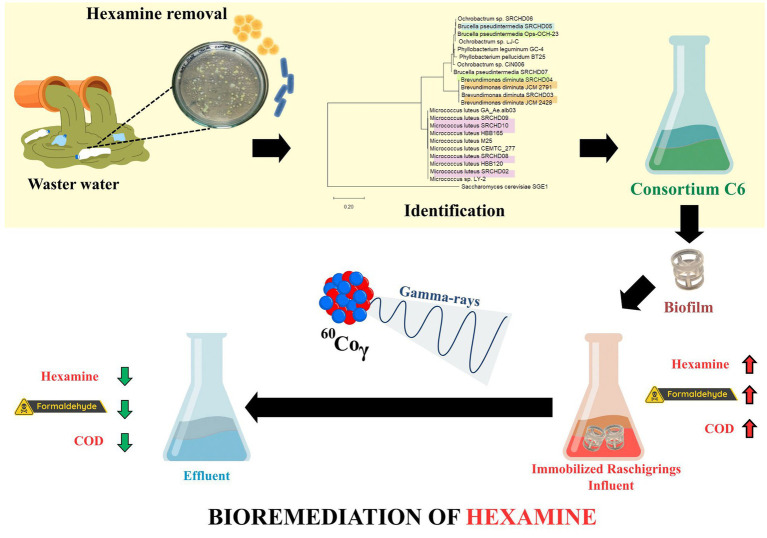

## Introduction

Industrial Revolution is strongly associated with the utilization of chemicals and the production of several types of pollutants. Aquatic ecosystems are continuously being contaminated. These pollutants have impacts at the micro to macro levels of the ecosystem’s organization. Nature has its own strategies for addressing pollutants through bioremediation, requiring adequate time for detoxification. The amount of waste generated continuously increases, minimizing the time available for detoxification and increasing pollutant concentration, thereby reducing the natural detoxification efficiency. The situation is often worsened by the presence of other pollutants with antimicrobial and ecotoxic properties. Most of the manufacturing industrial pollutants are listed as persistent organic pollutants (POPs) (Akhtar et al., 2021). POPs have harmful effects on the food chain, pose serious health hazards, and cause environmental ecotoxicity (Alharbi et al., 2018). Plastic industries began in the 1920s, and products made from plastic became regular necessities of life. Various studies reported that worldwide plastic production was between 310 and 359 million metric tons in 2016 and 2018, respectively (Plastics Europe, 2019). Asia has become a potential center for the receipt and production of plastic waste, leading to the generation of 121 million tons (Mt) of plastic waste from industrial and municipal sources (Liang et al., 2021). Various organic compounds (such as Polyethylene, organic alkenes) are combined with plastic to increase quality, flexibility, and longevity (Evode et al., 2021). Hexamine is commonly used in food packaging and plastic industries because of its antimicrobial and supportive features in the polymeric architectures, resulting in its leaching into the natural ecosystem during the degradation of plastic into microplastic (Devlieghere et al., 2000; Gogoi et al., 2024). Hexamine itself is a hydrophilic organic pollutant, which contributes to chemical oxygen demand (COD) (Gogoi et al., 2024) addition to wastewater coming from plastic and food industries (being a food preservative) (Liu et al., 2022). Hexamine has been utilized since the 18th century as an alternative to frequent antibiotic treatment for urinary tract infections (UTIs), helping to reduce the danger of emerging antimicrobial resistance (Nadeem and Hashim, 2024). Hexamine, as a pollutant, is significant because of its hydrophilic properties and by-products. In acidic conditions, hexamine breaks down into ammonia and formaldehyde, both of which play an important role in water pollution. This being a reversible reaction, leads to the production of hexamine in the presence of formaldehyde and ammonia in wastewater, hence emphasizing the rapid removal of both formaldehyde and ammonia from wastewater. Ammonia is listed as a major pollutant directly linked to eutrophication in natural ecosystems. Formaldehyde is used in the textile, food industries, furniture, and paint industries (Talaiekhozani et al., 2013). Formaldehyde is listed as a carcinogenic compound (International Agency for Research on Cancer, 2006). In the fish and meat industries, formalin is commonly used as a preservative. In the poultry sector, formaldehyde spray is used as a feed additive and as a surface sterilizer for eggshells (Williams, 1970). Based on the rapid and large-scale utilization of formaldehyde and its detrimental impact on the environment, it has become a big challenge and has attracted the attention of the scientific community for a better and more rapid removal (Guimarães et al., 2012). Similar to other organic pollution removal techniques, various physicochemical methods are available for removing formaldehyde, including activated carbon-based adsorption, which benefits from its porous structure, but it finds little practical application due to recovery and reusability issues (Satyam and Patra, 2024). New-generation photocatalytic oxidation methods are known for their reliance on costly compounds and the installation of expensive photoreactors (Cuerda-Correa et al., 2019). Advanced oxidation processes can completely oxidize organic compounds, producing carbon dioxide and water molecules in the presence of free radicals and oxidizing agents. Bioremediation is the key regulator for the natural cleaning of organic pollutants and the recycling of nutrients.

Microbial wastewater technologies efficiently remove organic pollutants, antimicrobial compounds, and heavy metals, and recycle water to the natural environment. The advanced biological technologies offer recovery of valuable by-products like biogas, bioethanol, and recovery of nutrients (Singh et al., 2024) to meet Sustainable Development Goals (SDGs). Researchers are continuously reporting microbial technologies for the treatment and production of economically valuable by-products (animal feeds and organic fertilizer). Halder et al. (2020) reported rapid conversion of dairy wastewater into liquid biofertilizer; this process efficiently removed 41.83% nitrate and 45.83% of phosphate with a 16-h hydraulic retention time (HRT). Application of this biofertilizer in mung beans could increase 2.6-fold in seed production (Halder et al., 2020). The process on further scale up could achieve the biotransformation of dairy wastewater into liquid biofertilizer within 4-h HRT at 11,000 L/day processing capacity with 77% nitrate, 36.4% phosphate, 62.8% protein, 26.3% BOD, and 39.5% COD reduction (Gogoi et al., 2021a,b). The biofertilizer was tested with a positive impact on 17 different varieties of economic crops. Doki et al. (2024) reported a 2,500 L wastewater treatment plant with 80–90% removal of COD (from 1,560 mg/L COD, 88 mg/L of ammonia, and 12.3 mg/L of phosphate), in batch mode operation with 120 HRT. Biswas et al. (2021) reported the use of dairy wastewater for the production of protein and carbohydrate-rich algal biomass via surface-attached growth, producing substrate for biofuel production with elimination of energy expenditure for algal harvesting. This process involved a two-step operation with bacterial treatment of 20-h followed by algae–bacterial incubation of 48-h. Biswas et al. (2022) reported microbial consortium-based bacterial biofilm reaction as a moving bed biofilm reactor for petrochemical wastewater treatment with 18-h HRT at 12,000 L/day processing capacity at East India Petroleum Private Limited. This was a sludge-free system that could recover the vast volume of waste for landscaping and firefighting purposes. These advanced microbial technologies are capable of meeting 11 SDGs out of 17 SDGs, that is, ~65% (Obaideen et al., 2022). These SDGs include SDG 1 and SDG 8 (advance treatment process offer income through recovery of valuable products, recycling of waste water to agricultural application) (Gogoi et al., 2022), SGD 2 (zero hunger, through the production of animal and aquaculture feeds, which support food chain and food security), SDG 3, and SDG 14 (Good health and wellbeing, life below water through efficient waste water treatment technologies reduce human health risk factors and protection of natural environment), SDG 6, SDG7, and SDG 13 (clean water and sanitation, affordable and clean energy, and climate action, through the effective removal of organic pollutants, generation of green energy, recycling of water, nutrients, and energy, and reduction of greenhouse gases production), SDG 9 (industry, innovation, and infrastructure through development of advance and real waste water treatment plants).

Biological treatment of hexamine-containing wastewater faces a major bottleneck due to the presence of antimicrobial compounds (formaldehyde), leading to associated sludge production. Removing sludge is expensive for an industrial setup. Therefore, the presence of hexamine and formaldehyde in wastewater become a major obstacle to meeting the SDGs. Formaldehyde targets structural and functional biomolecules in microbial cell walls and irreversibly binds to the primary growth-regulating molecules, including DNA, RNA, and proteins (Klein et al., 2022). Hexamine and its by-products, formaldehyde and ammonia, are common pollutants in various types of wastewaters, including sewage treatment plants (STPs) (residential, industrial, nuclear medicine centers, and cancer hospitals). Sewage from nuclear medicine centers (NMCs) is particularly complex due to the presence of low levels of radioactivity in addition to contaminants that are normally present. The presence of high amounts of ammonia and formaldehyde in wastewater can form hexamine (Gogoi et al., 2024; Hutnan et al., 2005; Samal et al., 2024). As per available norms of environmental protection agencies [International Atomic Energy Agency (IAEA), 2010], the prescribed discharge limits for radioactive effluents to the aquatic route are 0.02 mSv/a [Atomic Energy (Safe Disposal of Radioactive Wastes) Rules, 1987; Atomic Energy Regulatory Board (AERB), 2007]. Hence, wastewater treatment must be completed within the radioactive environment before discharge. The coexistence of radioactivity and chemical components, such as formaldehyde, poses a significant challenge for conventional treatment systems, underscoring the urgent need for integrated approaches capable of addressing such complex effluents. Bibliometric analysis of the last 5 years’ (2019–2024) data (VOSviewer version 1.6.20) showed a total of 143 publications on Radioactive waste bioremediation, 90 publications on hexamine removal from wastewater, while only 7 (Gogoi et al., 2024; Sharma and Patel, 2016; Gupta and Mittal, 2016; Middelhoven and van Doesburg, 2007; Hutnan et al., 2005; Bodik et al., 1991; Drtil et al., 1991) are on hexamine bioremediation, and none concerning remediation under radioactive background. Therefore, the development of an efficient, hexamine-degrading microbial formulation using well-characterized isolates is essential to enhance pollutant removal and maintain the effectiveness of wastewater treatment systems in meeting the SDGs, particularly in sensitive environments. Hence, this study reports the development of a radio-tolerant bacterial formulation for efficient removal of COD and hexamine through the removal of formaldehyde using well-characterized natural bacterial isolates.

## Materials and methods

### Strain characterization

The well characterized pure bacterial isolates obtained through serial dilution on the 50 mg/L hexamine-AS plate [1% tryptone (Himedia RM 029-500G, HiMedia Laboratories Pvt. Ltd., Mumbai, India), 0.5% yeast extract (Himedia RM 027-500G, HiMedia Laboratories Pvt. Ltd., Mumbai, India), and 1.5% agar (Himedia RM 026-500G, HiMedia Laboratories Pvt. Ltd., Mumbai, India), pH 7] (Samal et al., 2024) were grown in AS broth for 24, 48, and 72 h at 37 °C. The cell-free supernatant (centrifugation at 10,000 *g* followed by filtration through a 0.22 μm Whatman filter, Catalog No: 9913-2502) was used for COD (Gogoi et al., 2024), hexamine (Balasubramanian et al., 2003), formaldehyde (Nash, 1953), and ammonia (Gautam, 2011) quantification as per standard protocols. The extent of removal was determined by comparing the above concentration with that of un-inoculated medium. The COD of the solution was measured as per standard procedure (Gogoi et al., 2024) using the Equation 1:


(1) CODconcentration(mg/L)=(K×Abs+β)

where *K* = 2,249, Abs is the absorbance at 605 nm, and the value of *β* (interface) is 0.

The percentage of COD reduction following incubation was calculated as per Equation 2:


(2) $$\begin{array}{l} \text{Percentage of reduction}=\\ \frac{\text{Initial concentration}\;(0\;h)-\text{Final concentration}\;}{\text{Initial concentration}\;\mathrm{at}\;0\;h\;}\times 100 \end{array}$$

In aqueous solution, free formaldehyde and hexamine-bound formaldehyde were quantified based on Nash’s principle. The spectrophotometric quantification of formaldehyde was done using Nash’s reagent method (Nash, 1953). Nash’s reagent (ammonium acetate and acetylacetone) reacts with formaldehyde under neutral conditions, producing 3,5-diacetyl-1,4-dihydrolutidine. This compound is light-sensitive and has a maximum absorbance at 410 nm. The sample (cell-free supernatant) and reagent were mixed in a 1:1 ratio in a glass test tube manually, heated to 60 °C for 15 min, then incubated at room temperature in the dark for 30 min before measuring absorbance at 410 nm spectrophotometrically (Biobase BK-UV1000, BIOBASE Bioindustry (Shandong) Co., Ltd., Jinan, Shandong, China). The percentage of formaldehyde reduction was calculated as per Equation 2.

In the modified Hantzsch esters method (Balasubramanian et al., 2003), hexamine was quantified through hydrolysis under acidic conditions and continuous heating. Hexamine is hydrolyzed into ammonia and formaldehyde under mildly acidic conditions. The produced formaldehyde reacts with acetylacetone and ammonium ions to form Hantzsch ester in aqueous solution. The heating ensures maintaining formaldehyde as a monomer and reduction of the solubility of ammonia in water, thereby maintaining the mild acidic condition. In a 15 mL test tube, 2 mL of regent [0.3% (v/v) acetyl acetone and 0.2% acetic acid in 2.08 M ammonium acetate], 0.2 mL sample (cell-free supernatant) in 1.8 mL phosphate buffer (0.1 M at pH 6.0) was added, mixed manually, heated at 100 °C for 60 min in a water bath, incubated in dark at room temperature for 30 min before the absorbance was measured at 410 nm in a spectrophotometer (Biobase BK-UV1000, BIOBASE Bioindustry (Shandong) Co., Ltd., Jinan, Shandong, China). The percentage of hexamine reduction was calculated as per Equation 2.

Nessler’s reagent method (Gautam, 2011) was used to quantify ammonia. In this method, to remove turbidity of cell-free supernatant (5 mL), 10% (w/v) zinc sulfate (0.05 mL), and 0.025 mL of 6 N sodium hydroxide solution were added, mixed, and allowed to stand for 40 min at room temperature for flocculation. The produced flocks were removed through filtration using Whatman Grade 1 filter paper (GE Healthcare UK Limited, Buckinghamshire) (Catalog No. 1001125). In the next step, 0.05 mL of 50% (w/v) potassium sodium tartarate was used for the removal of interfering agents, which might adversely impact the accuracy of the assay (Utomo et al., 2022), followed by the addition of 0.06 mL of Nessler’s reagent (Himedia R010, HiMedia Laboratories Pvt. Ltd., Mumbai, India). The solution was mixed manually, and the absorbance of the solution was taken at 410 nm after 10 min of incubation at room temperature. The change in ammonia concentration was calculated as per Equation 2.

### Doubling time determination

The 96-well plate reader, with high-throughput growth evaluation via optical density measurement, followed by colony-forming unit (CFU/mL) determination using the spread plate method at two time points during the log phase of growth, provided doubling times for isolates using Equation 3. For the experiment 1% of actively growing culture of the individual isolate [SRCHD03 (1.94 × 10^5^ CFU/mL), SRCHD04 (2.51 × 10^6^ CFU/mL), SRCHD05 (8.36 × 10^5^ CFU/mL), SRCHD06 (5.4 × 10^5^ CFU/mL), and SRCHD07 (5.4 × 10^6^ CFU/mL)] was aseptically inoculated in 0.25 mL of AS broth in 96 well microtiter plate, placed inside a microplate spectrophotometer (Bio Tek, EPOCH2TS, Bio Tek Instruments, Inc., Winooski, USA) for 3 days at 37 °C with measurement of optical density at 30-min interval during the entire period. For convenience of representation, the data were plotted at 60-min interval. Doubling time of isolates was calculated using the formula given below:


(3) $$G=\frac{t}{3.3\;\log\;b/B}$$

In this formula, *G* = doubling time, *t* = time interval in min, *B* is the initial number of bacterial cells at the beginning of the time interval, and *b* is the final number of bacterial cells at the end of the time interval (Gogoi et al., 2024).

### Biofilm-forming ability

The principle of cell permeability of Crystal Violet (CV) stain was used for quantitative detection of the biofilm via spectroscopy. To 2-mL microcentrifuge tubes containing 0.6 mL of sterile AS media, 1% of an actively growing pure culture was inoculated and maintained at 37 °C under stationary conditions for 24 h (Martín Rosique et al., 2008). To it, 0.15 mL of 1% CV solution (Himedia S012-125 mL, HiMedia Laboratories Pvt. Ltd., Maharashtra, India) was added, incubated for 10 min for staining, and then washed 3 times with distilled water (at 10-min intervals) to remove unabsorbed stains. Decolorization of the stained biomass was done using 0.6 mL of 95% ethanol. The absorbance of the solution was recorded at 620 nm to determine the extent of biofilm formation.

### Phylogenetic analysis of the isolates

For the molecular characterization of the isolates, an outsourcing facility (GeneOmbio Technologies Pvt. Ltd., Maharashtra, India) was used for partial 16S ribosomal DNA (rDNA) sequencing. The phylogenetic tree was constructed from the obtained DNA sequence using MEGA version 12.1 software to understand relationships among the isolates and their closest neighbors.

### Characterization of the developed consortium

Based on their hexamine, formaldehyde, and COD removal ability, five isolates were selected for consortium development (Halder et al., 2020). The combinations tested are detailed in Table 1. The consortia were cultured in AS broth (containing 50 mg/L of hexamine) at 37 °C, and sampled at 24, 48, and 72 h, respectively. The cell-free supernatant was obtained as mentioned earlier under strain characterization. The filtrate was used for the quantification of COD (Gogoi et al., 2024), hexamine (Balasubramanian et al., 2003), formaldehyde (Nash, 1953), and ammonia (Gautam, 2011) quantification as per the standard protocols. As mentioned for the pure isolates, the growth curve, doubling time, and biofilm-forming ability of the most efficient consortium were assessed following 1% inoculum (7.9 × 10^5^ CFU/mL) in AS broth.

**Table 1:** Different combination of the isolates for consortia development.

SL No.	Combination of the isolates	Consortium
1	SRCHD05: SRCHD06 (1:1)	C1
2	SRCHD03: SRCHD05: SRCHD06 (1:1:1)	C2
3	SRCHD04: SRCHD05: SRCHD06 (1:1:1)	C3
4	SRCHD05: SRCHD06: SRCHD07 (1:1:1)	C4
5	SRCHD03: SRCHD04: SRCHD05: SRCHD06: SRCHD07 (1:1:1: 1:1)	C5
6	SRCHD02: SRCHD03: SRCHD04: SRCHD05: SRCHD06: SRCHD07 (1:1:1: 1:1:1)	C6

### Impact of low-dose γ irradiation on consortium performance

The purpose of the development of this consortium would be to use it in a low-radiation background STP in the future. To do so, the selected consortium was tested for tolerance to radiation. The dose selection was made taking into consideration that the consortium should be able to perform its function while operating in a radioactive environment. The lowest available dose of ^60^Co γ-rays at the irradiation facility (Board of Radiation & Isotope Technology–Department of Atomic Energy [BRIT-DAE] GC-5000 unit at Jadavpur University, Kolkata, India) was 188 times the permitted dose for discharge. Hence, the efficiency of the consortium for hexamine, COD, and formaldehyde removal upon incubation at ambient temperature was assessed, following irradiation with 3.75 Gray (Gy) and 5.25 Gy ^60^Co γ-rays. The 24 h-old culture and biofilm on polypropylene Raschig rings in sterile Falcon tubes (two conditions) were irradiated at the requisite doses in triplicate. Cultures were similarly maintained under unirradiated conditions as a positive control for both cases, while uninoculated medium was used as a negative control. After irradiation, 1% of the suspended culture (both irradiated and un-irradiated) was inoculated in fresh medium at 120 rpm shaking revival under ambient conditions for cell. The remaining cultures were harvested, and the cell-free supernatant was processed for biochemical analysis (hexamine, formaldehyde, and COD). Cultures were re-inoculated every 48 h to maintain and study the revival of the irradiated culture. This process was repeated for up to 8 rounds of re-inoculation (after every 48 h). In the case of biofilm, the medium was replaced in the Falcon tube every 48 h, and the change in concentration of COD, hexamine, and formaldehyde of the medium was assessed for each round of medium replacement from cell-free supernatant.

### Statistical analysis

The above-mentioned experiments were performed with at least three biological replicates, each with three statistical replicates (a total of nine replicates). The statistical validation of the data was carried out using an *F*-test followed by a *t*-test at the 95% confidence level (Microsoft Excel, 2007).

## Result

### Strain characterization

#### Phylogenetic analysis, biofilm forming ability, and doubling time of the isolates

**Figure 1:**
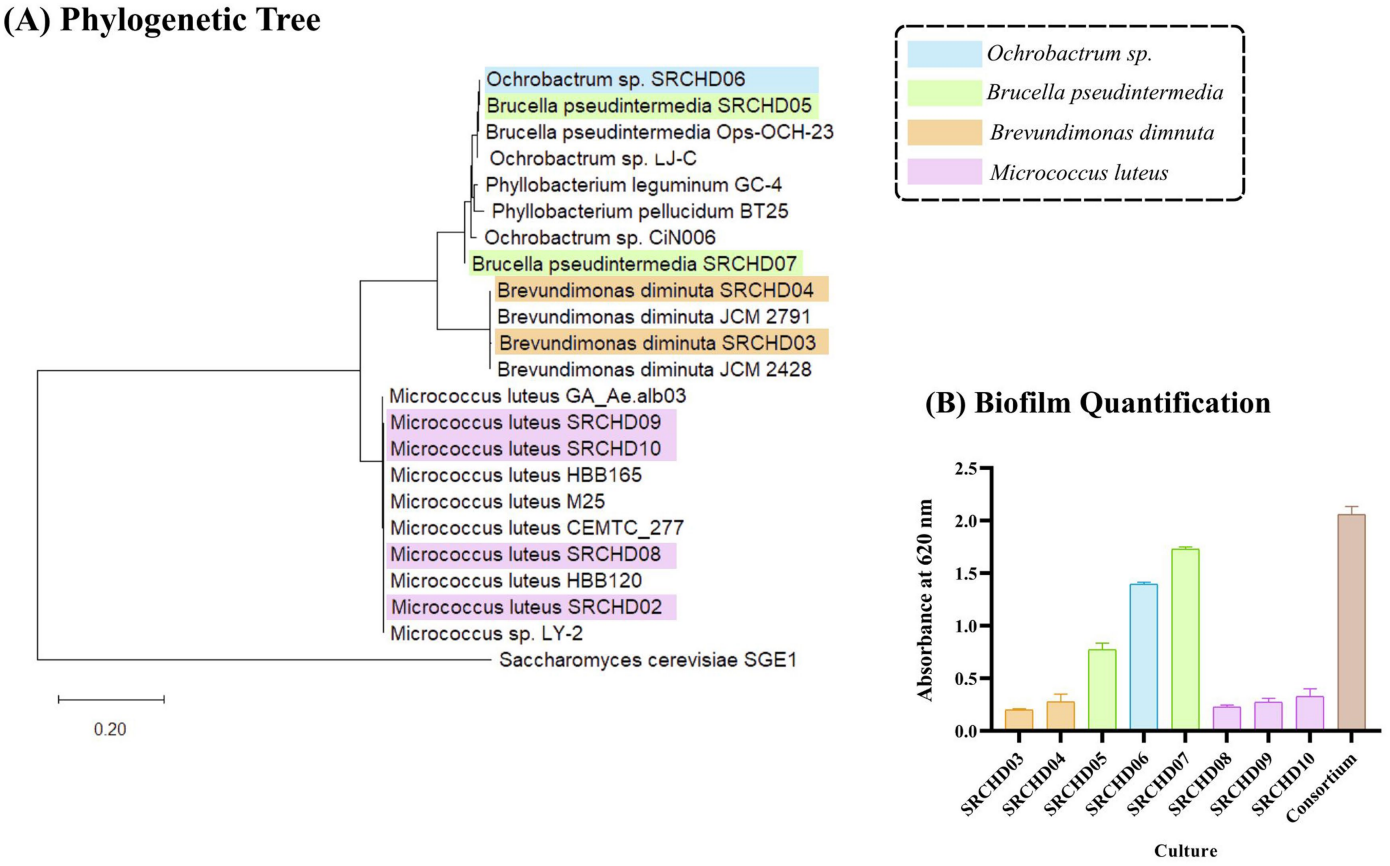
**(A)** Phylogenetic tree of isolates drawn using MEGA version 12.1 software based on the neighbor-joining method; **(B)** biofilm quantification of the isolates and consortium 6. All the experiments were conducted in biological triplicate. The statistical analysis was done through an *F*-test followed by a *t*-test at a 95% confidence level.

Partial 16S rRNA sequencing-based phylogenetic analysis revealed the formation of three clusters among the isolates with their closest neighbors (Figure 1A). Isolates SRCHD03, SRCHD04, SRCHD08, SRCHD09, and SRCHD10 were structured biofilm formers, and isolates SRCHD05, SRCHD06, and SRCHD07 were strong biofilm former (Figure 1B).

**Figure 2:**
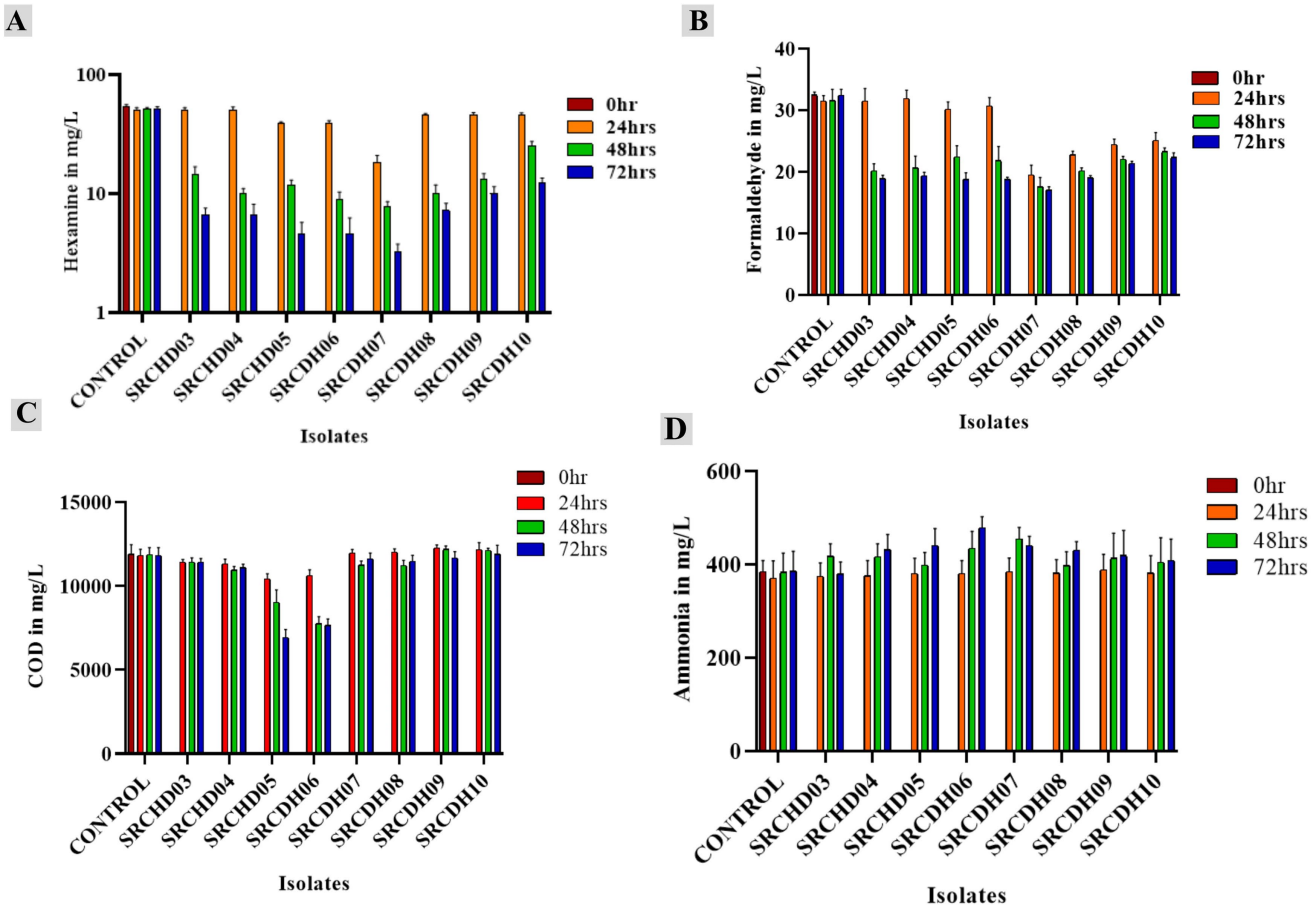
Bar graph representation of COD, hexamine, and its by-products (ammonia and formaldehyde) in suspended culture in AS medium at different time points: **(A)** hexamine quantification in mg/L from suspended culture of the isolates at 0, 24, 48, and 72 h, respectively; **(B)** formaldehyde quantification in mg/L from suspended culture of the isolates at 0, 24, 48, and 72 h, respectively; **(C)** COD quantification in mg/L from suspended culture of the isolates at 0, 24, 48, and 72 h, respectively; **(D)** ammonia quantification in mg/L from suspended culture of the isolates at 0, 24, 48, and 72 h, respectively. All the experiments were conducted in biological triplicates. The statistical analysis was done through an *F*-test followed by a *t*-test at a 95% confidence level.

Based on the above bioremediation data (Figure 2), five isolates (SRCHD03, 04, 05, 06, and 07) were selected for consortium development. The direct hexamine quantification data revealed all isolates to be efficient in removing hexamine after 48 h of incubation, while isolate SRCHD07 showed more than 66% reduction at 24th h of incubation (Figure 2A), indicating rapid removal of hexamine from the very beginning. This could be due to a higher number of cells per unit volume during the growth phase and can be confirmed by growth curve analysis. The isolate SRCHD07 showed the highest formaldehyde reduction (38%) at 24 h of incubation, followed by SRCHD08, SRCHD09, and SRCHD10 (30.72, 25.40, and 23.21, respectively). Overall, the 48 h of incubation showed a major reduction in the majority of the cases (Figure 2B). COD analysis revealed that isolates SRCHD05 and SRCHD06 were more efficient among the selected isolates. They could reduce COD by 42 and 36% after 72 h of incubation at 37 °C from an initial load of 11,900 mg/L, respectively (Figure 2C). Only SRCHD03 showed complete removal of produced ammonia (from the breakdown of hexamine into ammonia and formaldehyde) at 72 h of incubation. The remaining isolates showed an increase in ammonia concentration as an indication of hexamine degradation, but demonstrated an inability to remove ammonia (Figure 2D).

**Figure 3:**
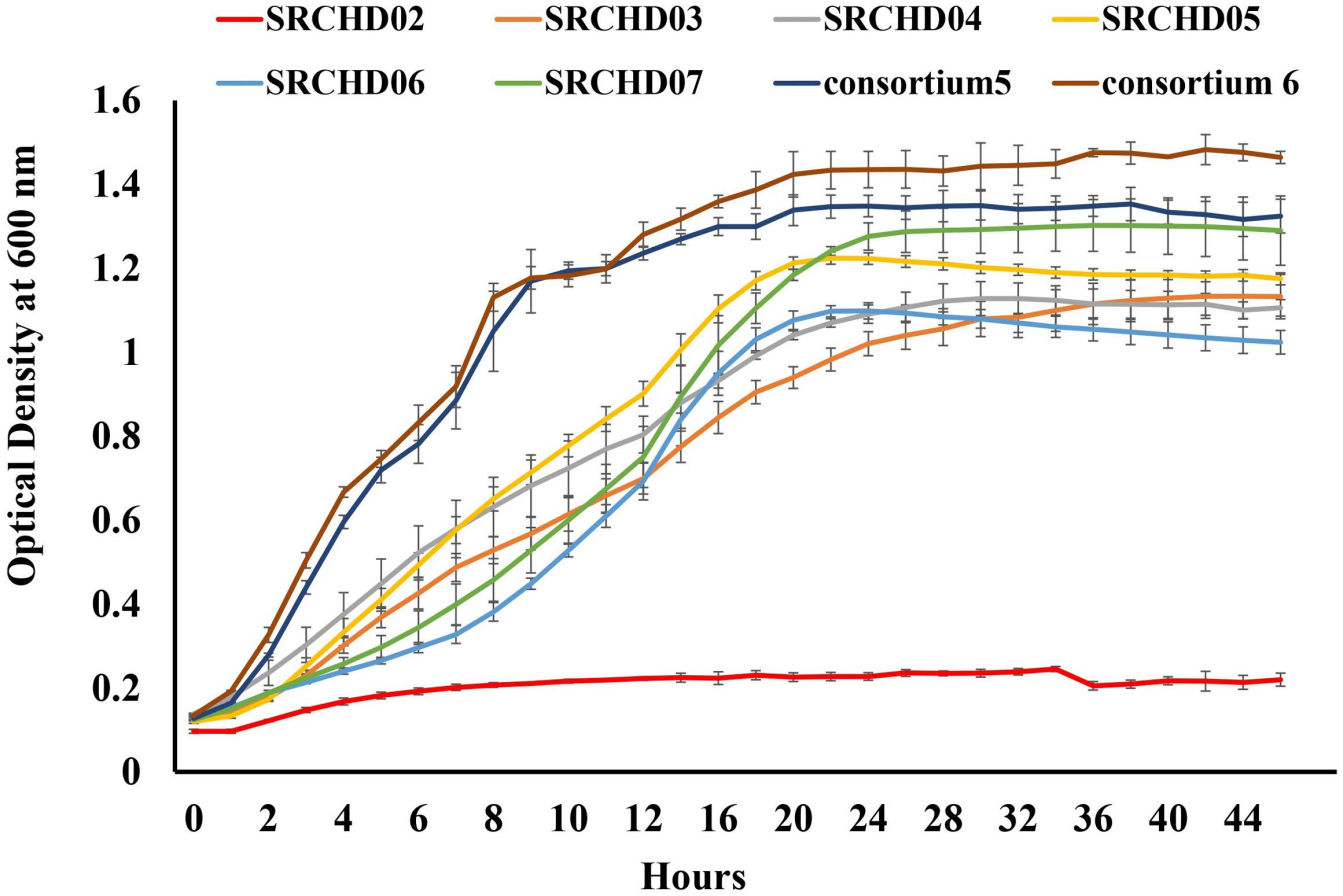
Growth curve of the isolates and consortium 5 and consortium 6.

The growth curve data of the selected isolates revealed that the isolate SRCHD07 was the fastest growing strain among the selected five isolates, with a log phase of 12 h and doubling time of 49 min and 6 s. Isolates SRCHD06 (14 h log phase, 128 min and 12 s doubling time), SRCHD05 (13.5 h log phase, 73 min and 2 s doubling time), SRCHD03 (20 h log phase, 121 min and 4 s doubling time), and SRCHD04 (25 h log phase, 80 min, and 6 s doubling time) took longer time to reach growth saturation (Figure 3).

#### Characterization of the developed consortium

Among the different consortia developed (Table 1), consortium (C5) showed 94.1% hexamine reduction associated with 42.4% COD and 49% formaldehyde reduction after 72 h of incubation, while producing ammonia simultaneously. Upon further modification with the addition of isolate SRCHD02 (consortium C6), the hexamine bioremedial ability increased further to 95.2% with 46.53% COD reduction associated with 50% formaldehyde reduction at 72nd h of incubation (Table 2). Growth curve of consortium C6 showed synergistic activity among the isolates, resulting in higher cell counts (Figure 3) and a reduced doubling time (40 min and 5 s, 4 h log phase) and shorter lag phase (1 h). The consortium was a strong biofilm former (Figure 1B).

**Table 2:** Hexamine degrading ability of the consortiums based on direct hexamine, formaldehyde, COD, and ammonia quantification.

Consortium	Hexamine reduction (percentage)			Formaldehyde reduction (percentage)			COD reduction (percentages)			Ammonia production (percentage)		
	24 h	48 h	72 h	24 h	48 h	72 h	24 h	48 h	72 h	24 h	48 h	72 h
C1	49.10	68.18	86.74	38.46	44.12	45.10	2.03	2.41	19.86	5.90	16.11	18.89
C2	64.20	77.26	89.07	37.12	41.69	41.57	7.39	18.85	26.86	15.71	21.64	23.51
C3	67.21	79.70	88.07	37.43	41.63	42.05	8.71	14.63	27.30	18.13	21.24	22.66
C4	71.13	83.62	91.11	42.11	43.76	45.64	4.81	14.26	19.61	19.98	23.67	24.63
C5	79.02	87.59	94.18	16.43	37.91	43.88	1.15	36.21	42.17	1.94	24.08	29.45
C6	84.24	91.96	95.26	34.34	48.10	49.23	7.78	38.86	46.48	3.89	19.54	28.57

#### Impact of low-dose γ irradiation on consortium performance

**Figure 4:**
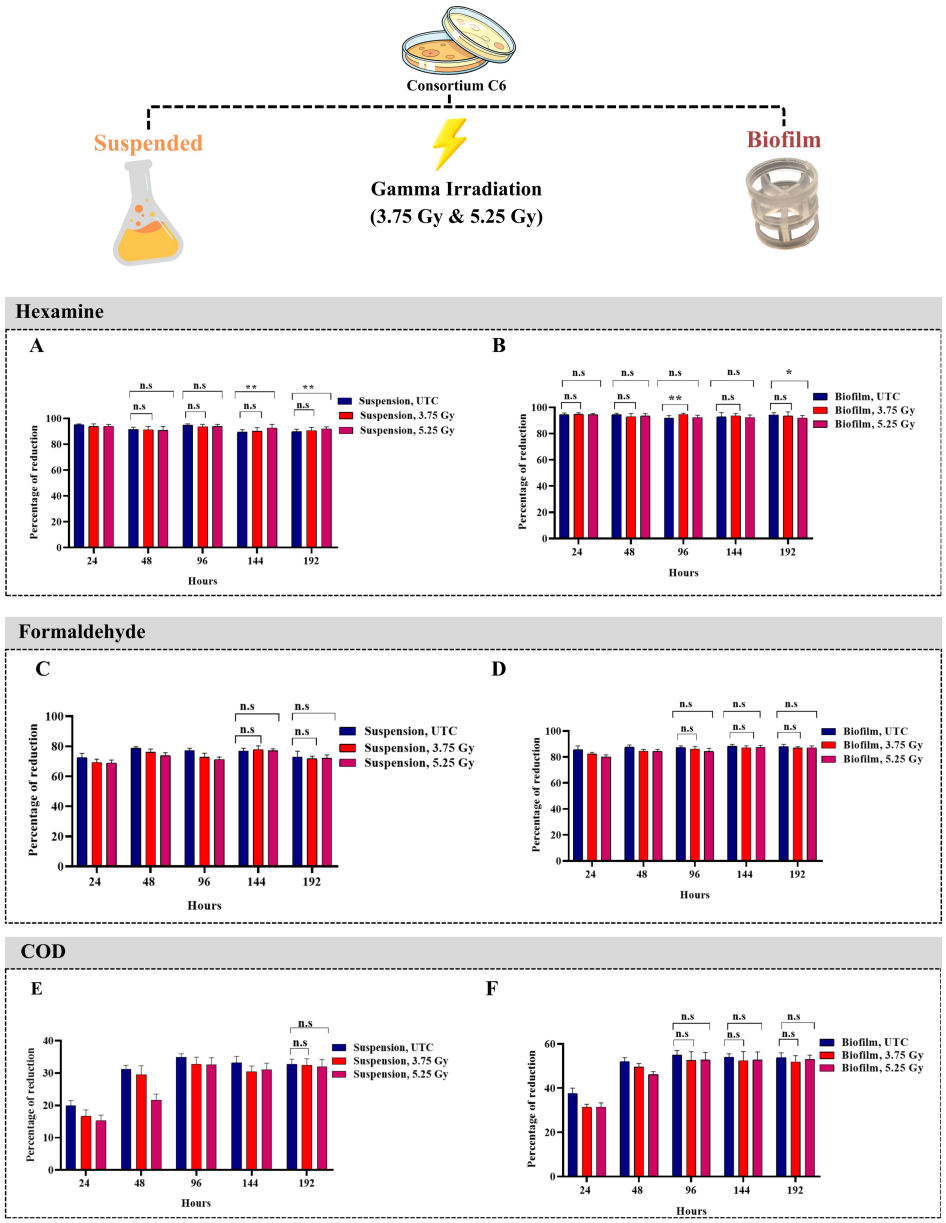
Bar graph representation of hexamine, formaldehyde and COD in suspended and biofilm culture in AS medium with 100 mg/L hexamine at different time points after low doses of gamma irradiation (3.75 Gy and 5.25 Gy) and untreated cultures (UTC). Statistically non-significant difference (n.s.) has *p* value > 0.05; lower reduction of the treated culture compared to UTC with statistically significant difference (*) having *p* value < 0.05; higher reduction of the treated culture compared to UTC with statistically significant difference (**) having *p* value < 0.05; **(A)** Hexamine quantification in mg/L from suspended culture; **(B)** Hexamine quantification in mg/L from biofilm culture: **(C)** formaldehyde quantification in mg/L from suspended culture; **(D)** formaldehyde quantification in mg/L from biofilm culture. **(E)** COD quantification in mg/L from suspended culture; **(F)** COD quantification in mg/L from biofilm culture. All the experiments were conducted in biological triplicate. The statistical analysis was done through an F-test followed by a T-test at a 95% confidence level.

The consortium C6 (un-irradiated) could remove 95.21% (*p*-value 2.2 × 10^−16^) hexamine (from 50 mg/L) after 24 h of incubation at ambient temperature. The reduction dropped to 93.78% (*p*-value 3.5 × 10^−2^) and 93.91% (*p*-value 1.5 × 10^−2^) following 3.75 and 5.25 Gy of ^60^Co γ irradiation, respectively. There was an insignificant impact of a higher dose (5.25 Gy) of ^60^Co γ irradiation compared with a lower dose (3.75 Gy) of ^60^Co γ-irradiation on hexamine removal (*p*-value 0.07) at 95% confidence level. In 48 h, statistical validation of hexamine removal data showed a *p*-value between 0.26 and 0.4 for cultures of un-irradiated, 3.75 Gy, and 5.25 Gy ^60^Co γ irradiated cells, indicating revival from the impact of irradiation (Figure 4A). In case of the biofilm-based system, there was no change in performance in terms of Hexamine removal following irradiation (*p*-value between 0.29 and 0.5) even at 24th h of incubation (Figure 4B).

The optimal reduction of formaldehyde by the consortium C6 (un-irradiated) occurred at 48 h. It could remove 78.69% (*p*-value 2 × 10^−15^) formaldehyde (from 40.64 mg/L) at 48 h of incubation, which reduced to 76.02% (*p*-value 3.4 × 10^−3^) and 73.79% (*p*-value 3 × 10^−6^) following 3.75and 5.25 Gy of ^60^Co γ irradiation, respectively. The formaldehyde-removing ability was regained after 144 h post-irradiation with re-culturing every 48 h (un-irradiated 76.85%, 3.75 Gy 77.91%, 5.25 Gy 77.05%, with *p*-value ranging from 0.1 to 0.3) (Figure 4C). In the case of the biofilm-based system, the extent of formaldehyde removal was much higher than suspended culture, and the system was revived from the impact of irradiation with 96 h of incubation (*p*-value between 0.054 and 0.06) (Figure 4D).

The consortium C6 (un-irradiated) showed the optimal reduction in COD at 96 h of incubation. It could remove 34.94% (*p*-value 3.17 × 10^−20^) COD (from 13,367 mg/L), which reduced to 32.75% (*p*-value 9 × 10^−3^) and 32.53% (*p*-value 6.8 × 10^−3^) following 3.75and 5.25 Gy of ^60^Co γ irradiation, respectively. The COD removing ability was regained after 192 h post-irradiation with re-culturing every 48 h (un-irradiated 32.72%, 3.75 Gy 32.43%, 5.25 Gy 31.91%, p-value between 0.2 and 0.3) (Figure 4E). In the case of the biofilm-based system, the extent of COD removal was much higher than suspended culture, and the system was revived from the impact of irradiation by 96 h of incubation (*p*-value between 0.059 and 0.065) (Figure 4F).

## Discussion

The major industrial sectors, such as the plastic, pharmaceutical, phenolic resin, and rubber industries, have higher demand and greater economic impact on national growth and development. Generated wastewater from these industries with hexamine becomes detrimental to the natural environment. Advanced biological treatment technologies using efficient microbes represent a sustainable approach to treating industrial wastewater. Efficient and synergistic microbes in microbial formulations can achieve higher pollutant removal efficiency than individual isolates (Bhambri et al., 2025). In this study, consortium C6 consists of bacterial isolates from the following genera: *Brevundimonas* sp., *Brucella* sp.*, Ochrobactrum* sp., and *Micrococcus* sp. Bacteria from all these genera have been reported for the bioremediation of polycyclic aromatic hydrocarbons (PHAs). Li reported air purification through formaldehyde removal of microbial isolate *Ochrobactrum* sp. strain ZH-1 (Li et al., 2023). Jing reported 85.37% (from 5 mg/kg) degradation of polycyclic aromatic hydrocarbons (PHAs) with a consortium *of Brucella* sp., and *Cellulosimicrobium* sp. within 120 days (Jing et al., 2025). These microbes have been reported to be used for the bioremediation of antimicrobial compounds, such as Cephalexin (CEX). Kou reported *Brevundimonas* sp. CEF1 has the ability to degrade Cephalexin (CEX) at a rate of 94.26% within 8 h of incubation. These microbes could activate stress response mechanisms, cytochrome P450-mediated responses, and hydroxylate antimicrobial compounds (Kou et al., 2025). In this study, we have found that this consortium could remove hexamine, formaldehyde, and COD in both suspended and immobilized conditions. Different microbial isolates have been reported for the removal of hexamine and its by-products (formaldehyde and ammonia) (Table 3). Phylogenetic analysis (Figure 1A) shows the presence of *B. pseudintermedia* and *Ochrobactrum* sp. Based on the literature survey, *B. pseudintermedia* is being reported for the first time for hexamine and formaldehyde bioremediation. Literature reported the synergism of *Brucella* sp. with other bacterial isolates for efficient removal of organic pollutants (Jing et al., 2025), while it is the first report of *Ochrobactrum* sp. being involved in hexamine bioremediation. The isolates SRCHD05 and SRCHD06 showed higher COD reduction efficiency than the other isolates. Literature (Sharma and Patel, 2016; Gogoi et al., 2024) reports that the microbes from this genus are capable of breaking down synthetic organic compounds through the production of different classes of enzymes, such as hydrolases and oxidoreductases. Veeranagouda et al. (2006) reported the *Ochrobactrum* sp. DGVK1 was capable of degrading dimethylformamide (DMF), resulting in the release of dimethylamine and ammonia in the medium. The stain could utilize formaldehyde and formate as a carbon source.

**Table 3:** Reported microbe for formaldehyde and ammonia removal.

Bioremediation	Involved microbe	Efficiency of the treatment	Reference
Suspended culture	*Nannochloropsis oculata* ST-3 strain	99.3% of formaldehyde removal from 19.9 mg/L initial load, within 22 days of incubation	Yoshida et al. (2009)
Immobilized culture	*Pseudomonas putida*	90–95% formaldehyde reduction, within 12 h from the initial concentration of 370–377 mg/L	Zvulunov et al. (2019)
Biofiltration	*Pseudomonas* sp.	50% formaldehyde reduction after 4 days, the initial inlet concentration was 60 mg/L	Wang et al. (2019)
Suspended culture	*Bacillus amyloliquefaciens* sp.	95.8% of formaldehyde reduction from an initial concentration of 100 mg/L within 34 h	Han et al. (2020)
Biodegradation	*Klebsiella pneumoniae* TN-1	83.26% ammonia removal from 300 mg/L initial load within 7 days in mineral salt medium	Zhang et al. (2021)
Immobilized condition	*Bacillus albus* ASSF01	4.7 mg/L ammonia-containing effluents to 0.5 mg/L at 14th h of inoculation under ambient conditions in a sludge-free process.	Gogoi et al. (2021a,b)
Suspended culture	*Bacillus tequilensis* A2	Achieving 90% ammonia reduction from a 20 mg/L initial concentration after 72 h of incubation under optimized conditions	Wang et al. (2023)

Molecular mechanisms for hexamine and its by-products (formaldehyde and ammonia) utilization by the microbes will vary among community members. The hexamine-bound formaldehyde was utilized by the microbe as a carbon source (Gogoi et al., 2024; Sharma and Patel, 2016). Microbes utilize several cofactors (tetrahydrofolate, tetrahydromethanopterin, mycothiol, and glutathione) to uptake formaldehyde, detoxify it, and oxidize it to carbon dioxide with the production of energy (Yurimoto et al., 2005). Tolerance and stress-mediated cellular mechanisms depend on distinct cellular transcriptional regulatory sigma factors. Sigma factor SigH is important for the detoxification of formaldehyde (Wani and Jain, 2024). In *Paracoccus denitrificans*, formaldehyde reacts with glutathione to form S-hydroxymethylglutathione (Goenrich et al., 2002). In the next step of formaldehyde detoxification, several regulatory alcohol dehydrogenase enzymes are involved. Klein reported a novel formaldehyde dehydrogenase enzyme for the detoxification of formaldehyde in *Bacillus subtilis* (Klein et al., 2024). In assimilation pathways, formaldehyde interacts with sugar phosphates, entering several pathways, such as the ribulose monophosphate pathway, the xylulose monophosphate pathway, and the serine pathway (Yurimoto et al., 2005). In contrast, ammonia can be utilized by several aerobic and anaerobic microbes as a nitrogen source for biomass and energy production. Microbes can assimilate ammonia through several pathways, such as the glutamate dehydrogenase pathway in *Enterobacter* sp. B12 (Gao et al., 2024). In anaerobic conditions, microbes can convert ammonia to nitrogen gas. The metabolic activity of the ammonia-removal microbes is influenced by the surrounding environment. The literature reports that low dissolved oxygen can stimulate ammonia-oxidizing microbes’ ammonia-removal activity (Wang et al., 2025). The presence of hexamine-like complex organic pollutants in water can lower dissolved oxygen levels, which might further accelerate the microbe’s ammonia removal efficiency. In contrast, the nitrification of ammonia produces protons, which accelerate the hexamine hydrolysis (Gogoi et al., 2024). The degradation and removal efficiency of synthetic organic pollutants is reflected in the COD of the treated solution.

The doubling-time assessment in suspension culture helped explain the faster formaldehyde removal by isolate SRCHD07. The higher cell number of SRCHD07 at any given point in time, as compared to other isolates, results in higher bioremediation ability. *B. pseudintermedia* could utilize antimicrobial compounds as a sole nutrient source. Benaoura reported complete removal of the antibiotic cefazolin (from 400 mg/L) within 12 h (Benaoura et al., 2025). The bioremediation ability of *B. pseudintermedia* indicates its potential application in advanced biological wastewater treatment plants. This study focused on the biofilm-based bioremediation of hexamine. Literature reports advanced biological technologies for efficient wastewater treatment (Table 3). In new generation systems for wastewater treatment investigated by Moussavi et al. (2012), the investigating team used a two-step method, where advanced oxidation systems, specifically the electro-Fenton (EF) method, were employed for the complete degradation of a toxic organic pollutant (formaldehyde) at an initial concentration of 7,500 mg/L. This was followed by biological treatment (with a biomass-to-effluent ratio of 1:3) for COD removal. After 16 days of operation, the COD (initially 3,890 mg/L) in the effluent from the EF reaction was reduced to below 50 mg/L. Méndez et al. (2015) reported that, in the presence of peroxide, formaldehyde was removed from simulated wastewater containing phenol-formaldehyde using the hybrid Fenton and biological aerated filter (BAF) technique. This advanced method had a high removal capacity of phenol-formaldehyde, but the utilization of expensive chemicals, such as peroxide, with low efficiency for the reduction of the biotoxicity of formaldehyde was the reason behind its limitation. Formaldehyde-degrading microbe utilizes formaldehyde as a principal and intermediate substrate of carbohydrate metabolism (ribulose and xylulose monophosphate and ribulose bisphosphate carboxylase pathway) and protein metabolism (serine pathway) for biomass and energy production (Shao et al., 2020). *Ochrobactrum* sp. and *Brevundimonas diminuta* have been shown to harbor genes with functional interactions, which could be further detailed in a future study to understand the underlying molecular mechanism of bioremediation in the current context.

Microbes in immobilized conditions can perform better in bioremediation than in suspended conditions. The immobilized condition provides a well-established cellular network, metabolic exchanges, and adaptability to environmental fluctuations. Biofilm facilitates rapid absorption and degradation of pollutants compared to suspended culture (Mishra et al., 2022) through the establishment of the cellular network process, mostly due to higher biomass per unit area. The consortium showed stronger biofilm-forming ability than the individual isolates. The autoinducer-based signaling networks (mainly quorum sensing through Rhll/RhlR), have a role in industrial wastewater treatment by enhancing community survival. The microbes synthesize and secrete extracellular polymeric substances (proteins and heteropolysaccharides), aiding in rapid bioremediation (Chattopadhyay et al., 2022). Different types of autoinducers have a role in the biofilm formation that is used in the removal of toxic organic pollutants (Feng et al., 2013). In the biofilm system reported in this study, the consortium C6 could remove 94.41% hexamine 85.56% formaldehyde within 24 h of incubation, associated with 37.64% COD removal efficiency. The literature reports that quorum sensing and growth factors are the main factors behind the recovery of biofilm (Jing et al., 2019) following inhibitor exposure. The consortium C6 was the most efficient in terms of bioremediation, achieving hexamine, formaldehyde, and COD removal in 24, 48, and 96 h, respectively. Following irradiation with ^60^Co γ rays (an inhibitor in this case) under suspended conditions, the consortium took 48 h (hexamine removal), 144 h (formaldehyde removal), 192 h (COD removal) to regain its bioremediation ability. In the case of the biofilm-based system, there was no change in performance in terms of hexamine removal following irradiation (*p*-value between 0.29 and 0.5) even at 24th h of incubation. The formaldehyde- and COD-removing abilities were much higher in the biofilm-based system, with revival within 96 h of incubation.

Hence, the consortium was radio-tolerant, with the ability to rapidly revive. As the background radiation in NMC STP is much lower than the used dose, it is expected that the consortium will be able to perform bioremediation within such STPs. From the above data, it was revealed that the current consortium (C6) could remove 1.23 grams (g) of hexamine per kilogram (kg) of matrix (Gogoi et al., 2021a,b) within 24 h (1,230 mg/kg), which increased to 1.3 g/kg (1,300 mg/kg) after 192 h. Irradiation of the cells does not impact their ability to remove hexamine. The consortium could remove 0.92 g (920 mg) of formaldehyde per kg of matrix within 48 h of incubation. Efficiency following irradiation resumed within 192 h. The optimum COD reduction (192.96 g/kg or 192,960 mg/kg) was achieved within 96 h of incubation post-irradiation. The future scope of this study will include testing the consortium as an immobilized system with an ammonia-removing unit in series with simulated wastewater and effluent.

## Conclusion

This study reports the hexamine-removing ability of *B. pseudintermedia* and *Ochrobactrum* sp. A radio-tolerant, efficient hexamine-removing consortium, which functions under both suspended and immobilized conditions, was found to regain its bioremedial activity within 96 h of incubation after 3.75and 5.25 Gy of ^60^Co γ-irradiation upon immobilization. Hexamine and its by-product, formaldehyde, have a detrimental effect on native bioremedial microbes and advanced biological sewage treatment plants/effluent treatment plants (STPs/ETPs). Hence, this consortium, as a biofilm, might be a good alternative in the future for treating wastewater in STPs and ETPs using hexamine, thereby achieving SDGs through the application of microbial technology.

## Data Availability

The original contributions presented in the study are included in the article/supplementary material, further inquiries can be directed to the corresponding author.

## Glossary

COD: chemical oxygen demand
POP: persistent organic pollutant
Mt: million tonnes
UTI: urinary tract infection
EF: electro-Fenton
BAF: biological aerated filter
TFD: tylosin fermentation dreg
STP: sewage treatment plant
NMC: nuclear medicine center
IAEA: International Atomic Energy Agency
CFU: colony-forming unit
CV: crystal violet
g: gram
kg: kilogram
^60^Co: cobalt-60
γ: gamma
mSv/a: millisieverts per annum
SDGs: Sustainable Development Goals
CEX: Cephalexin
PHAs: polycyclic aromatic hydrocarbons
AERB: Atomic Energy Regulatory Board
HRT: hydraulic retention time
DMF: dimethylformamide
